# Epidemicity of cholera spread and the fate of infection control measures

**DOI:** 10.1098/rsif.2021.0844

**Published:** 2022-03-09

**Authors:** Cristiano Trevisin, Joseph C. Lemaitre, Lorenzo Mari, Damiano Pasetto, Marino Gatto, Andrea Rinaldo

**Affiliations:** ^1^ Laboratory of Ecohydrology ENAC/IIE/ECHO, École polytechinque fédérale de Lausanne (EPFL), Lausanne 1015, Switzerland; ^2^ Dipartimento di Elettronica, Informazione e Bioingegneria, Politecnico di Milano, Milano 20133, Italy; ^3^ Dipartimento di Scienze Ambientali, Informatica e Statistica, Università Ca’ Foscari Venezia, Venezia 30172, Italy; ^4^ Dipartimento ICEA, Università degli studi di Padova, Padova 35131, Italy

**Keywords:** effective reproduction numbers, next generation matrix, prognostic indicators, spatially explicit infection models, ranking emergency interventions

## Abstract

The fate of ongoing infectious disease outbreaks is predicted through reproduction numbers, defining the long-term establishment of the infection, and epidemicity indices, tackling the reactivity of the infectious pool to new contagions. Prognostic metrics of unfolding outbreaks are of particular importance when designing adaptive emergency interventions facing real-time assimilation of epidemiological evidence. Our aim here is twofold. First, we propose a novel form of the epidemicity index for the characterization of cholera epidemics in spatial models of disease spread. Second, we examine in hindsight the survey of infections, treatments and containment measures carried out for the now extinct 2010–2019 Haiti cholera outbreak, to suggest that magnitude and timing of non-pharmaceutical and vaccination interventions imply epidemiological responses recapped by the evolution of epidemicity indices. Achieving negative epidemicity greatly accelerates fading of infections and thus proves a worthwhile target of containment measures. We also show that, in our model, effective reproduction numbers and epidemicity indices are explicitly related. Therefore, providing an upper bound to the effective reproduction number (significantly lower than the unit threshold) warrants negative epidemicity and, in turn, a rapidly fading outbreak preventing coalescence of sparse local sub-threshold flare-ups.

## 1. Introduction

Much work on the mathematical description of unfolding cholera outbreaks has been put forth in the aftermath of the disastrous Haiti epidemic, which started in 2010 [1–3]. Cholera is an infectious waterborne disease caused by contamination with *Vibrio cholerae*, a bacterium that colonizes the intestine after ingestion and can cause, if untreated, severe dehydration and electrolyte loss resulting at times in the death of the host. As infected individuals shed pathogens through their faeces, high bacterial loads may reach communities’ water supplies and fuel the infection cycle in settings where basic sanitation is lacking.

One of the most severe cholera outbreaks in recent history struck Haiti just months after a catastrophic earthquake had hit the country on 12 January 2010, imposing a major death toll and effectively destroying whatever sanitation and transportation infrastructures pre-existed. Sadly, cholera—at that time unknown to the island for more than two centuries—was accidentally seeded into the country from Nepal, where a severe cholera outbreak had been ongoing [2,4]. Given the lack of basic sanitation infrastructure and the obvious unpreparedness of the population, cholera quickly propagated through the whole country showing remarkable spatial signatures [1,2,4–10]. In 2013, the Haitian health authorities, jointly with public and private partners, teamed up to establish a protocol for eradicating the disease from Haiti [2]. The chosen strategy consisted of non-pharmaceutical interventions (NPIs), such as targeted water, sanitation and hygiene (hereafter WaSH) interventions, deployed intensively and in a capillary manner [2], combined with a vaccination campaign of large proportions—and difficult logistics [9,11]. WaSH interventions were carried out by rapid response teams depending on an alert system and involved, e.g. educational sessions for the local population, distribution of chlorination and sanitation products, nursing support and additional prophylaxis. These interventions sought to curb the persistence of *V. cholerae* in the environment by reducing local and global exposure to contaminated waters, and halting the shedding of extremely high and initially hyperinfectious bacterial loads from infectious individuals into the aquatic reservoirs, where pathogens can survive long enough to close the transmission cycle [3,12,13]. Vaccination protocols and deployment had to be adapted in time, e.g. because of extreme events like hurricane Matthews ravaging the southwest of Haiti [14].

Here, we move from a detailed understanding of the effects of each WaSH intervention carried out in space and time in Haiti, to investigate whether reliable prognostic indices exist that would allow a team of on-field epidemiological experts to rapidly decide the best interventions at any given stage of an unfolding cholera epidemic. To that end, in this work, we shall adapt an extensively used family of spatio-temporal epidemiological models [3,5,6,14–19] to account for the effect of WaSH interventions. This containment effect is connected to a decrease in the rates governing the exposure to the infection and the contamination of water reservoirs, which in turn affect the reproduction number (the number of secondary infections caused by an infected individual, which may be derived by a variety of methods [20–24]). In a naive population (i.e. lacking any prior immunity), the basic reproduction number $R_{0}$ discriminates between a spreading epidemic eventually leading to endemic transmission ($R_{0}>1$) and a short-lived outbreak asymptotically waning towards a disease-free equilibrium ($R_{0}<1$). Analogously, effective reproduction numbers $R_{t}$ are employed when susceptible individuals do not comprise the entire population, and/or time-dependent containment measures or environmental forcing are accounted for [3,23,25]. Therefore, in the present work, we shall also establish and compute conditions for possible long-term circulation of the pathogen either in uncontrolled settings ($R_{0}$), or else when containment efforts are deployed ($R_{t}$), using a next-generation matrix approach suitable to tackle spatial systems like those where human mobility is a significant driver of infections [26–29].

It has been noted, however, that both $R_{0}$ and $R_{t}$ overlook an epidemiological system’s short-term transient response to a perturbation of its state (say, the local injection of a number of infectious individuals through human mobility). Whatever the long-term attainment of disease-free conditions, the nature and the extent of local transient responses following a perturbation may influence the geography of an unfolding outbreak. For instance, local subthreshold flare-ups may have severe consequences in terms of death toll, hospital capacity or economic damages, and in terms of coalescence of local outbreaks [29,30]. Generalized reactivity analysis, a recently developed methodological framework for the study of transient dynamics in ecological systems subject to external perturbations [3], may thus be applied [29–33]. Generalized reactivity focuses on the relative contribution of the state-space components of a metapopulation or metacommunity disease ecology model to transient behaviour following an impulsive perturbation. It can be used to define a threshold-type quantity, termed the epidemicity index. Here, we shall study a proper formulation of such index for cholera models, where the need to characterize the ecological reservoirs of the bacterial component poses interesting mathematical challenges. In fact, generalized reactivity analysis requires the definition of a proper eco-epidemiological output transformation of the relevant state variables, whose transient dynamics is then determined by the dominant eigenvalue of a suitable Hermitian matrix obtained from the Jacobian associated with the equilibrium and the system output transformation [29,30,33]. Here, we shall investigate and apply strategies for the computation of the epidemicity index proxied by the effective reproduction number as a prognostic tool to complement the design of emergency intervention strategies. Our results may find applications in the design of emergency interventions facing the kind of trade-offs typical of decision-making on alternative strategies for containment measures during unfolding cholera outbreaks.

## 2. Methods

### 2.1. Data sources

Weekly incidence data to calibrate the model were gathered from the epidemiological bulletins provided by the Haitian Ministry for Public Health and Population [34] and are available at a departmental level. Population distribution has been obtained through the Institut Haïtien de Statistique et d’Informatique. Non-pharmaceutical interventions (NPIs) data are available from the literature [9]. The information about all interventions deployed, originally available on a municipal and weekly scale, has been geographically upscaled to the departmental level and temporally downscaled (assuming a constant intervention rate) at the daily scale. Rainfall data have been gathered from two sources. Measurement up to March 2015 come from the TRMM 3B42 RT Derived Daily Product (https://disc2.gesdisc.eosdis.nasa.gov/dods/TRMM_3B42RT_Daily_7.info), while subsequent data were gathered from GPM satellite-based precipitation measurements (https://disc.gsfc.nasa.gov/).

### 2.2. A cholera modelling framework

We adopt a spatially explicit SIRBV compartmental model progressively improved and verified [5,6,14,15,18,30,35,36] that studies the demography of the disease within local communities compartimentalized into classes depending on their health status. In each community *i*, we specifically identify the coupled rates of change of: susceptibles (*S*_*i*_); symptomatic infected individuals (*I*_*i*_) (an individual who has contracted cholera and is currently shedding pathogens at a rate exceeding six stools per day); asymptomatic infected individuals (*A*_*i*_) who have contracted cholera but do not show its clinical manifestations; recovered individuals (*R*_*i*_), who developed acquired immunity for a given time span following either symptomatic or asymptomatic infection. Because the cholera vaccine is administered in two doses, each compartment is split into three different cohorts, namely unvaccinated, once-vaccinated and twice-vaccinated individuals. This additional stratification makes it possible to account for differential vaccine efficacy depending on the number of doses received (e.g. [37]). WaSH interventions (local measures aimed at safe water distribution and the enforcement of preventive epidemiological measures of public health; e.g. [1,2,4]) are also accounted for in view of the importance of their deployment in the Haiti outbreak.

The spatial structure of the model is described by a network, where the nodes are the 10 Haitian administrative departments and the links represent mobility matrices for healthy (i.e. mobile) individuals. We consider that a fraction *m* of the population is actually mobile and may become exposed to pathogens not only in their home community but also when travelling. We need to model the probability that a traveller originating from a community, say *i*, will reach another community, say *j*, as destination, and to this end gravity or radiation models are used (see [3,38,39]). Here, because of the spatially heterogeneous population density within Haiti [3], we relied on a gravity model by quantifying the normalized flux *Q*_*ij*_ of mobile susceptibles from node *i* to node *j* as $Q_{ij}=H_{j}\;e^{-d_{ij}/D}/[\sum_{k\neq i}H_{k}\;e^{-d_{ik}/D}]$, where *H*_*j*_ is the population of the destination community, *d*_*ij*_ is the distance between origin and destination communities (properly determined depending on the prevailing transportation means) and *D* is a scaling parameter that can be estimated through model fitting. The mobility model has been firstly defined on small geographical units corresponding to the Haitian watersheds [5,6,15] and later upscaled to the departmental level proportionally to the resident population [14].

In addition to 12 epidemiological compartments, we further implemented a supplementary departmental compartment representing the bacterial concentration in the local aquatic environment (*B_i_*), which effectively drives the spread of the epidemic as contagion is usually contracted by ingesting a body mass-dependent dose of pathogens [3,6,35,40]. The abundance of the pathogen in the environment is assumed to decay exponentially at rate *μ*_*B*_ and replenished proportionally to the current number of infected *I*_*i*_. The compartment of susceptibles is supplied by a constant recruitment term *μH*_*i*_, where *μ* is the baseline human mortality rate (affecting equally all other human compartments of the model in the given *i*th node) and *H*_*i*_ is the population size at demographic equilibrium. Key to the descriptive power of the model is the formulation of the force of the infection [6,19,35], describing the rate at which susceptibles are exposed to pathogens

2.1 $$F_{i}(t)=(1-m)\beta_{i}(t)\frac{B_{i}}{B_{i}+1}+m\sum_{\;j\neq i}Q_{ij}\beta_{j}(t)\frac{B_{j}}{B_{j}+1},$$

where we allow infection for both the non-mobile (1 − *m*) fraction of the population, which can only get infected locally, and the mobile fraction of the population (*m*), which may contract the pathogen also while travelling. The force of infection depends on the local exposure rate *β*_*i*_(*t*), which reflects the probability per unit time that susceptible individuals come into contact with contaminated water, and the instantaneous local pathogen abundance in the *i*th compartment, *B*_*i*_. The bacterial compartment is scaled and computed as $B_{i}=B_{i}^{⋆}/K$ where $B_{i}^{⋆}$ is the actual bacterial concentration in the local population in the *i*th department and *K* is the half saturation constant, the concentration that would yield a 50% chance of becoming infected if exposed to it. It is also assumed that the aquatic reservoir volume is directly proportional to the abundance of resident people [6,40]. The latter is included in the force of infection through a logistic response curve [40]. At the Haitian departmental scale, hydrological transport ruling the spread of pathogens through river networks may be neglected [15]. Also, based on previous analyses, a commonly adopted specific compartment of exposed individuals *E* [8] has been ruled out by formal model comparison via the Akaike criterion [6]. The weekly recorded infections in week ending on day *t* are computed as $C_{i}(t)=\int_{t-7}^{t}\sigma F_{i}(\tau )S_{i}(\tau )\;d\tau$, being *σ* the symtomatic fraction of newly infected. NPIs, such as WaSH actions, as well as reports of recent disease incidence in a territory, may condition large-scale public behaviour, thereby resulting in altered exposure patterns. We model nodal NPIs as a rate of instantaneous interventions deployed and we denote it as NPIs_*i*_(*τ*) being *i* the community where NPIs are deployed. Clearly, weekly deployed (i.e. reported) interventions are ${\mathrm{WaSH}}_{i}(t)=\int_{t-7}^{t}{\mathrm{NPIs}}_{i}(\tau )\;d\tau$. We assume, after some preliminary computations unreported here, that the exposure rate *β*_*i*_(*t*) decreases following an exponential law after the occurrence of many cases or after NPIs interventions:

2.2 $$\begin{array}{r} \beta_{i}(t)=\beta_{0}\exp[-\frac{1}{H_{i}\psi }\int_{t-t_{0}}^{t}\sigma F_{i}(\tau )S_{i}(\tau )\;d\tau \\ -{\left(\int_{t-t_{w}}^{t}\frac{{\mathrm{NPIs}}_{i}(\tau )}{H_{i}}\left(1-\frac{t-\tau }{t_{w}}\right)\;d\tau \right)}^{\xi_{1}}], \end{array}$$

where *t*_0_ represents the memory of public perception of the infection risk, *ψ* is a parameter scaling the past disease incidence with the nodal population *H*_*i*_, *t*_*w*_ is the duration of NPIs' effectiveness, and *ξ*_1_ is a shape parameter. NPIs are weighted linearly so that their efficacy ranges between 1 at the time the intervention is deployed and 0 when the given NPI has been deployed since *t*_*w*_ days. The two integrals are numerically approximated with a standard rectangular integration method, with step equal to 1 day. We normalized the disease in incidence and the NPIs with the nodal population to guarantee homogeneity across units. In addition, detected symptomatic infections *C*_*i*_(*τ*) and NPIs_*i*_(*τ*) are neglected with *τ* < 0 as they refer to pre-outbreak time-points. Susceptible individuals becoming infected at rate $F_{i}(t)$ can either develop symptoms (a fraction *σ* of those exposed) or not (fraction 1 − *σ*). Symptomatic individuals can die because of the illness at rate *α* or recover at rate *γ*. They contribute to onward cholera transmission by shedding *V. cholerae* bacteria into the local aquatic reservoir at a rainfall-enhanced rate [6,8,41]. We hypothesize that pathogen shedding by infected individuals is also affected by NPIs, namely that the larger the amount of recent sanitary interventions, the lower the shedding rate. The shedding rate thus reads

2.3 $$\theta_{i}(t)=\theta_{0}[1+\phi J_{i}(t)]\exp\left[-{\left(\int_{t-t_{w}}^{t}\frac{{\mathrm{NPIs}}_{i}(\tau )}{H_{i}}\left(1-\frac{t-\tau }{t_{w}}\right)\;d\tau \right)}^{\xi_{2}}\right],$$

where *J*_*i*_(*t*) is the incoming rainfall, *θ*_0_ is the scaled number of bacteria shed per quota of the total resident population, *ϕ* is the rainfall contamination enhancement parameter and *ξ*_2_ is a shape parameter. NPIs are weighted in the same way as in (2.2) and similar numerical integration techniques are employed. Asymptomatic individuals shed less bacteria (say, by a reduction factor *r* < 1) than symptomatic and clear the infection at rate *γ*. Recovered individuals obtain temporary immunity to cholera infection before becoming susceptible again at rate *ρ*. All individuals not showing clinical signs of cholera infection (i.e. susceptible, asymptomatic and recovered people) are considered eligible for the vaccine. The vaccination rates are calculated as

2.4 $$\nu_{i}^{d}(t)=\frac{\text{first doses distributed in node }i\text{ on day }t}{S_{i}(t)+R_{i}(t)+A_{i}(t)}$$

and

2.5 $$\nu_{i}^{dd}(t)=\frac{\text{second doses distributed in node }i\text{ on day }t}{S_{i}^{1}(t)+R_{i}^{1}(t)+A_{i}^{1}(t)}$$

for first and second doses, respectively. One or two doses of vaccine are assumed to effectively protect from infection only a fraction $\eta_{i}^{d}(t)$ and $\eta_{i}^{dd}(t)$ of the recipients, respectively. Superscripts of the epidemiological compartments *S*, *A* and *R* denote the number of vaccine doses received.

To summarize, in each node *i*, our epidemiological model is governed by the following set of ordinary differential equations:

2.6a $${\dot{S}}_{i}=\mu (H_{i}-S_{i})-F_{i}(t)S_{i}-\nu_{i}^{d}(t)S_{i}+\rho R_{i}$$

2.6b $${\dot{I}}_{i}=\sigma F_{i}(t)S_{i}-(\gamma +\alpha +\mu )I_{i}$$

2.6c $${\dot{A}}_{i}=(1-\sigma )F_{i}(t)S_{i}-\left[\gamma +\mu +\nu_{i}^{d}(t)\right]A_{i}$$

2.6d $${\dot{R}}_{i}=\gamma (I_{i}+A_{i})-\left[\mu +\rho +\nu_{i}^{d}(t)\right]R_{i}$$

2.6e $${\dot{S}}_{i}^{1}=\nu_{i}^{d}(t)S_{i}-F_{i}(t)\left[1-\eta_{i}^{d}(t)\right]S_{i}^{1}-\left[\mu +\nu_{i}^{dd}(t)\right]S_{i}^{1}+\rho R_{i}^{1}$$

2.6f $${\dot{I}}_{i}^{1}=\sigma F_{i}(t)\left[1-\eta_{i}^{d}(t)\right]S_{i}^{1}-(\gamma +\alpha +\mu )I_{i}^{1}$$

2.6g $${\dot{A}}_{i}^{1}=(1-\sigma )F_{i}(t)\left[1-\eta_{i}^{d}(t)\right]S_{i}^{1}+\nu_{i}^{d}(t)A_{i}-[\gamma +\mu +\nu_{i}^{dd}(t)]A_{i}^{1}$$

2.6h $${\dot{R}}_{i}^{1}=\gamma (I_{i}^{1}+A_{i}^{1})+\nu_{i}^{d}(t)R_{i}-\left[\rho +\mu +\nu_{i}^{dd}(t)\right]R_{i}^{1}$$

2.6i $${\dot{S}}_{i}^{2}=\nu_{i}^{dd}(t)S_{i}^{1}-F_{i}(t)\left[1-\eta_{i}^{dd}(t)\right]S_{i}^{2}-\mu S_{i}^{2}+\rho R_{i}^{2}$$

2.6j $${\dot{I}}_{i}^{2}=\sigma F_{i}(t)\left[1-\eta_{i}^{dd}(t)\right]S_{i}^{2}-(\gamma +\alpha +\mu )I_{i}^{2}$$

2.6k $${\dot{A}}_{i}^{2}=(1-\sigma )F_{i}(t)\left[1-\eta_{i}^{dd}(t)\right]S_{i}^{2}(t)+\nu_{i}^{dd}(t)A_{i}^{1}-(\gamma +\mu )A_{i}^{2}$$

2.6l $${\dot{R}}_{i}^{2}=\gamma (I_{i}^{2}+A_{i}^{2})+\nu_{i}^{dd}(t)R_{i}^{1}-(\rho +\mu )R_{i}^{2}$$

2.6m $${\dot{B}}_{i}=-\mu_{b}B_{i}+\theta_{i}(t)\frac{I_{i}^{P}(t)}{H_{i}}$$

**Figure 1.**
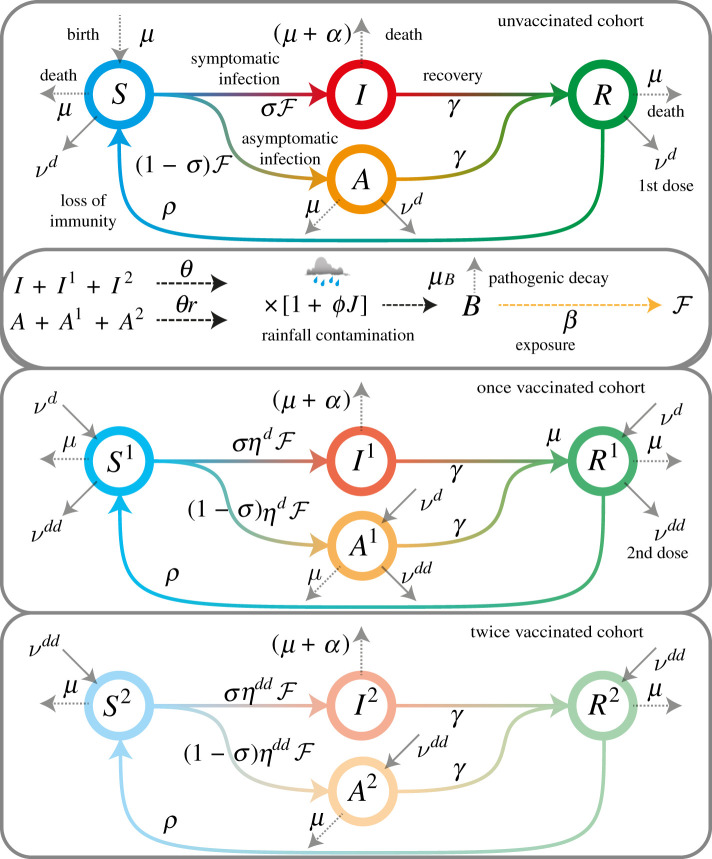
Outline of the model compartments and their interactions.

2.6n $$\mathrm{and}\;I_{i}^{P}(t)=I_{i}^{t}(t)+r\left[(1-m)A_{i}^{t}(t)+m\sum_{\;j\neq i}Q_{\;ji}A_{j}^{t}(t)\right],$$

where: *i* = 1, *N* indexes the *N* = 10 Haitian departments considered here; and $X_{i}^{t}(t)=X_{i}(t)+X_{i}^{1}(t)+X_{i}^{2}(t)$, *X* ∈ {*I*, *A*}, is the total abundance of infected people, either symptomatically (*X* = *I*) or asymptomatically (*X* = *A*), regardless of their vaccination status. See the electronic supplementary material for additional details. The scheme of the model is illustrated in figure 1.

### 2.3. Parameter calibration

We employed the DREAM_ZS_ algorithm [42,43] to calibrate the model against empirical data (i.e. reports of local weekly disease incidence). The algorithm implements several (in our case, three) Markov chains that efficiently explore the parametric hyperspace and converge to the posterior distribution. As we have no prior knowledge regarding the posterior distribution, we initialize the algorithm with a broad flat uniform distribution for each of the parameters. For the sake of simplicity, we implemented the negative residual sum of squares as our objective function, see electronic supplementary material. Specifically, we calibrate our model in two phases. Initially, all parameters unrelated to WaSH interventions are fitted between 20 October 2010 and 28 June 2013. The parametric set that maximizes the objective function is then applied to the whole period, while WaSH parameters are calibrated between 28 June 2013 and 30 June 2017.

**Figure 2.**
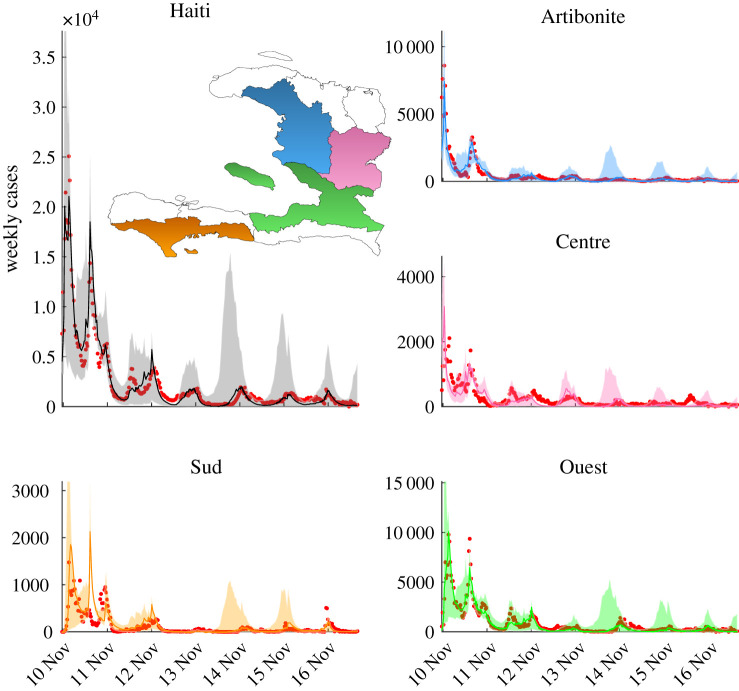
Model fit against the observed data (red dots) for the whole national area (top left) and four selected departments. The solid line is the best-fit simulation, while the coloured ribbons show the 50% confidence intervals due to parameters’ uncertainty.

All Markov chains (see also electronic supplementary material) converged to a stable posterior distribution, and the best fit yielded a Nash–Sutcliffe efficiency (see [6] and electronic supplementary material) of 0.851 throughout the entire simulated time span. The calibration was stopped when the Gelman–Rubin convergence diagnostic index remained steadily below the threshold of 1.05 (electronic supplementary material). Table 1 shows the parametric set that endows the model with the best fit of the computed and observed cholera incidence in the 10 Haitian departments, while figure 2 shows the best fit obtained for the simulation. The chosen model is able to reproduce all epidemic peaks due to the variety of disease-revamping mechanisms, in particular those due to intense tropical rainfall washing out open-air defaecation sites, which are the norm in Haiti [36]. The increase in exposure rates resulting from the loss of cautious behaviour that may typically follow a low-incidence period [6] was also considered. Finally, including NPIs into the adopted model allows one to properly reproduce the actual real-time dynamics of the Haitian cholera outbreak. In this way, in fact, the model can take into account not only the dynamics of the infection but also human interventions aiming to stem the propagation of cholera in Haiti. This is therefore an improvement of results that were obtained by earlier versions of this model (see e.g. [19]) that did not explicitly consider WaSH efforts nor hypothesized a shorter duration of acquired correct behaviour following a period when cholera transmission was rather sustained.

**Table 1.** List of model parameters, their prior distribution and values associated with the best fit.

definition	symbol	prior	value	units
**referenced parameters**				
life expectancy	1/*μ*	—	61.4 [44]	year
recovery rate	*γ*	—	0.2 [6]	day^−1^
cholera fatality rate	*α*	—	0.004 [16]	day^−1^
**baseline model parameters**				
baseline shedding rate	*θ*_0_	[0; 10]	0.336	day^−1^
mobile share	*m*	[0; 1]	0.152	—
scaling distance	*D*	[0; 500]	1.873	km
rainfall contamination	*ϕ*	[0; 20]	0.069	day mm^−1^
loss of immunity rate	*ρ*	[0; 1]	0.018	day^−1^
symptomatic share	*σ*	[0; 0.3]	0.022	—
pathogenic decay	*μ*_*B*_	[0; 1]	0.135	day^−1^
baseline exposure rate	*β*_0_	[0; 10]	6.643	day^−1^
prevalence scaling	*ψ*	[0; 10]	0.080	—
memory duration	*t*_0_	[8; 985]	941	day
asymptomatic shedding reduction	*r*	[0; 1]	5.21 × 10^−5^	—
**NPI-specific parameters**				
scaling exponent for exposure	*ξ*_1_	[0; 5]	0.160	—
scaling exponent for shedding	*ξ*_2_	[0; 5]	0.095	—
NPIs effectiveness duration	*t*_*w*_	[0; 365]	235	day

**Figure 3.**
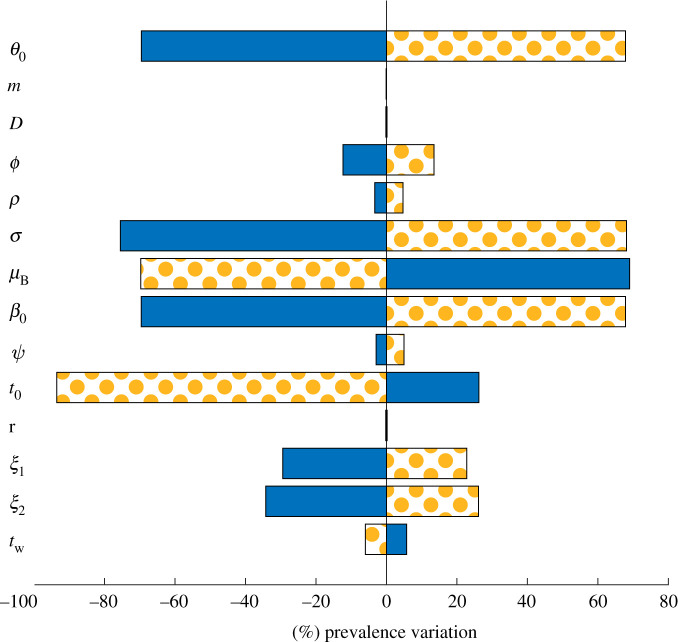
Sensitivity analysis for the calibrated model. Parameters were perturbed once at a time, each with a ±10% variation. The figure illustrates the per cent changes of the total reported number of simulated cases over the second calibration period (July 2013 to June 2017) with respect to the best-fit results. Solid blue or orange dot-filled bars correspond to negative or positive variations of the model parameters, respectively.

The calibrated parameters suggest that around 15% of the population may be considered mobile to the effects of disease spread, thus contributing to cross-contamination between different departments. To better grasp the influence of each parameter on the simulated number of total recorded cholera infections, we performed a sensitivity analysis (figure 3) where each parameter was perturbed with a ±10% variation. The most sensitive parameters proved to be the symptomatic fraction of new infections *σ*, the pathogens’ natural mortality *μ*_*B*_, the baseline exposure rate *β*_0_ and the baseline shedding rate *θ*_0_. The model is also sensitive to the duration of the acquired correct behaviour *t*_0_.

The calibration of the epidemiological model was deemed satisfactory, as the model reproduces reasonably well the epidemic unfolding—even when figures become small and a stochastic modelling framework might have been more appropriate [3]. The uncertainty range is computed by resampling the posterior distributions as in [14] (see electronic supplementary material). An increase in uncertainty can be observed around early July 2013, due to the resampling of the posterior distributions of the NPI parameters. The loss of immunity rate equals 0.018 day^−1^, corresponding to a mean duration of acquired immunity slightly exceeding two months, which is lower than the values commonly reported by WHO, which attest post-symptomatic infection immunity to around 3 years [45]. However, this parameter averages between symptomatic and asymptomatic infections, as asymptomatic infections confer a shorter post-recovery immunity and only 2.2% of the simulated infections are symptomatic. This figure, which is substantially lower than estimates such as [46], could be explained by misreporting of symptomatic or pauci-symptomatic individuals, which is not explicitly accounted for in this model.

### 2.4. Stability of disease-free equilibrium and next-generation matrices

Our derivation follows the approach traced by a number of relevant contributions [26,30,33,35,47,48]. Specifically, we evaluate our key epidemiological indices, i.e. the effective reproduction number and the epidemicity index, in a temporal and spatially explicit manner. The epidemicity index requires the specification of which states-at-infection (*sensu* [34]) are included to calculate the norm of the system output **y**. We are addressing the symptomatically infectious compartment with the output being defined as follows: [30,31]:

2.7 $$y(t)=\{I_{i}(t);I_{i}^{1}(t);I_{i}^{2}(t),\;i=1,\ldots ,10\},$$

whose norm is

2.8 $$\|y(t)\|=\sqrt{\|I(t){\|}^{2}+\|I^{1}(t){\|}^{2}+\|I^{2}(t){\|}^{2}}.$$

The asymptomatic infectious compartment is hereafter disregarded due to its estimated low shedding rate compared to the symptomatic compartment (see the value of parameter *r* in table 1), at a great simplification of the relevant algebra. In this way, the infectious subsystem, which is used for calculating the reproduction number (see below), is also restricted to the vector **y**. To further simplify the analysis of the model, we assume bacteria to hold faster dynamics than the other state variables. As such, we impose a local equilibrium condition

2.9 $$\frac{dB_{i}}{dt}=0,$$

which allows us to directly compute the bacterial concentration as a linear function of the infectious pool

2.10 $$B_{i}(t)=\frac{\theta_{i}(t)I_{i}^{P}(t)}{\mu_{B}H_{i}},$$

where $I_{i}^{P}$ is the total infectious pool of node *i* (see equation (2.6*n*)). The system shown in equations (2.6) can be easily rewritten by leaving out equation (2.6*m*). The force of infection can be rearranged accordingly

2.11 $$F_{i}(t)=(1-m)\beta_{i}(t)\frac{\theta_{i}(t)I_{i}^{P}(t)}{\theta_{i}(t)I_{i}^{P}(t)+\mu_{B}H_{i}}+m\sum_{\;j\neq i}Q_{ij}\beta_{j}(t)\frac{\theta_{j}(t)I_{j}^{P}(t)}{\theta_{j}(t)I_{j}^{P}(t)+\mu_{B}H_{j}}.$$

The derivative of the force of infection with respect to each of the symptomatically infected stages $I_{k}^{d}$ (*d* = 1, 2 are the doses of vaccine received by an infected individual) yields

2.12 $$\frac{\partial F_{i}(t)}{\partial I_{k}^{d}}=\left\{ \begin{array}{cc} (1-m)\beta_{i}(t)\frac{\theta_{i}(t)\mu_{B}H_{i}}{{\left[\theta_{i}(t)I_{i}^{P}(t)+\mu_{B}H_{i}\right]}^{2}}\; & \text{if }k=i\;\\ mQ_{ik}\beta_{k}(t)\frac{\theta_{k}(t)\mu_{B}H_{k}}{{\left[\theta_{k}(t)I_{k}^{P}(t)+\mu_{B}H_{k}\right]}^{2}}\; & \text{otherwise.}\; \end{array}\right.$$

One can thus compute the generalized Jacobian matrix that is associated with the modified ODE system along a given trajectory. Let *f*_*p*_ be the generic *p*th ODE equation and *X*_*q*_ the generic *q*th state variable. The elements *j*_*pq*_ of the Jacobian matrix **J** are defined as

2.13 $$j_{\;pq}=\frac{\partial f_{p}}{\partial X_{q}},$$

and **J** = [*j*_*pq*_]. To compute the dynamic disease reproduction number and the epidemicity index, one should only consider the infectious subsystem, which corresponds to the state variables pertaining to the infected stages of the model. The infectious subsystem **y** can be obtained from the algebraic transformation **y** = **Wx** of the full system state $x=\{S_{i},I_{i},\ldots ,R_{i}^{2},\;i=1,\ldots ,10\}$, with

2.14 $$W=\left(\begin{array}{cccccccccccc} 0_{n} & U_{n} & 0_{n} & 0_{n} & 0_{n} & 0_{n} & 0_{n} & 0_{n} & 0_{n} & 0_{n} & 0_{n} & 0_{n}\\ 0_{n} & 0_{n} & 0_{n} & 0_{n} & 0_{n} & U_{n} & 0_{n} & 0_{n} & 0_{n} & 0_{n} & 0_{n} & 0_{n}\\ 0_{n} & 0_{n} & 0_{n} & 0_{n} & 0_{n} & 0_{n} & 0_{n} & 0_{n} & 0_{n} & U_{n} & 0_{n} & 0_{n} \end{array}\right),$$

where **U**_**n**_ and **0**_**n**_ symbolize the identity matrix of size *n* and the square null matrix of order *n*, respectively. The dynamics of the infectious subsystem are thus described by the reduced-order Jacobian

2.15 $$\begin{array}{rl} J_{t}^{⋆} & =WJ_{t}W^{T}\\ & =\left(\begin{array}{ccc} \sigma {F^{\prime }}_{t}S-\Phi U_{n} & \sigma {F^{\prime }}_{t}S & \sigma {F^{\prime }}_{t}S\\ \sigma {F^{\prime }}_{t}(U_{n}-\eta^{d})S^{1} & \sigma {F^{\prime }}_{t}(U_{n}-\eta^{d})S^{1}-\Phi U_{n} & \sigma {F^{\prime }}_{t}(U_{n}-\eta^{d})S^{1}\\ \sigma {F^{\prime }}_{t}(U_{n}-\eta^{dd})S^{2} & \sigma {F^{\prime }}_{t}(U_{n}-\eta^{dd})S^{2} & \sigma {F^{\prime }}_{t}(U_{n}-\eta^{dd})S^{2}-\Phi U_{n} \end{array}\right), \end{array}$$

where *T* indicates matrix transposition; **F′** is the matrix whose terms are the derivatives of the force of infection as defined in equation (2.11); **S**, **S**^**1**^ and **S**^**2**^ are diagonal matrices whose non-zero elements represent the abundance of unvaccinated, once-vaccinated and twice-vaccinated susceptibles in the 10 Haitian departments; **η**^**d**^ and **η**^**dd**^ are diagonal matrices whose non-zero elements are the department-specific vaccine efficacies for first and second doses, respectively; and Φ=γ+α+μ.

### 2.5. Computation of the disease reproduction number

We adopt here the next-generation matrix method [47] to compute the effective reproduction number. Specifically, we introduce a transmission matrix **T**_**t**_ and a transition matrix $\Sigma_{t}$ so that their sum yields the Jacobian matrix of the system

2.16 $$J_{t}^{⋆}=T_{t}+\Sigma_{t}.$$

The transmission matrix includes the term related to the rate of appearance of new infections and is computed as

2.17 $$T_{t}=\left(\begin{array}{ccc} \sigma F^{\prime }S & \sigma F^{\prime }S & \sigma F^{\prime }S\\ \sigma F^{\prime }(U_{n}-\eta^{d})S^{1} & \sigma F^{\prime }(U_{n}-\eta^{d})S^{1} & \sigma F^{\prime }(U_{n}-\eta^{d})S^{1}\\ \sigma F^{\prime }(U_{n}-\eta^{dd})S^{2} & \sigma F^{\prime }(U_{n}-\eta^{dd})S^{2} & \sigma F^{\prime }(U_{n}-\eta^{dd})S^{2} \end{array}\right),$$

and the transition matrix contains the other infectious-related terms

2.18 $$\Sigma_{t}=\left(\begin{array}{ccc} -\Phi U_{n} & 0_{n} & 0_{n}\\ 0_{n} & -\Phi U_{n} & 0_{n}\\ 0_{n} & 0_{n} & -\Phi U_{n} \end{array}\right)=-\Phi U_{3n}.$$

One can thus define the next-generation matrix as

2.19 $$K_{t}=-T_{t}{\left(\Sigma_{t}\right)}^{-1}=-\frac{1}{\Phi }T_{t},$$

and finally obtain the effective reproduction number as the spectral radius P(·) of the next-generation matrix

2.20 $$R_{t}(t)=P(K_{t}).$$

### 2.6. Computation of the epidemicity index

Generalized reactivity regulates the tendency of a dynamical system, observed through a suitable output transformation of the system state, to temporarily amplify the effects of an impulsive perturbation to an otherwise asymptotically stable equilibrium [30,31]. A reactive transient behaviour is connected to the sign of the derivative of the norm of the system output (i.e. in our case, the infectious subsystem) being positive

2.21 $${\frac{d\|y\|}{dt}|}_{t=0}>0.$$

The above condition is verified when the largest eigenvalue (denoted as *λ*_max_(·)) of the Hermitian part of the reduced-order Jacobian (equation (2.15)) is positive

2.22 $$\lambda_{\max}(H(J_{t}^{⋆}))>0,$$

where the Hermitian matrix is defined as $H(J_{t}^{⋆})=(J_{t}^{⋆}+J_{t}^{⋆T})/2$. We define the epidemicity index as the largest eigenvalue of the aforesaid Hermitian matrix

2.23 $$e_{t}(t)=\lambda_{\max}(H(J_{t}^{⋆})).$$

A positive value of the epidemicity index thus denotes a short-term instability of the disease-free equilibrium, which indicates that epidemic flare-ups may develop following a perturbation even in the presence of an asymptotically stable steady state. On the contrary, a negative value of the epidemicity index ensures that all the eigenvalues of the Hermitian matrix associated with the infectious subsystem are negative, thus preventing transient epidemic spikes from happening. Box 1 further introduces the theoretical basis for a key development of this paper.

Box 1. **Derivation of the analytical relationship between the reproduction number and the epidemicity index.**

The reproduction number and the epidemicity index can be mathematically related through some algebraic manipulations. Let us now consider a model with a single unvaccinated cohort, out of simplicity. The infectious subsystem of the Jacobian matrix would read

2.24 $$J_{t}^{⋆}=\sigma F^{\prime }S-\Phi U_{n},$$

where **F′** is defined in equation (2.12). The transmission matrix is defined as

2.25 $$T_{t}=\sigma F^{\prime }S,$$

while the transition matrix is simply defined as follows:

2.26 $$\Sigma_{t}=-\Phi U_{n}.$$

The assumption that the infectious pool is much smaller compared to the whole population of a given node *i* ($I_{i}^{P}\ll H_{i}$) allows simplification of equation (2.12) as follows:

2.27 $$\frac{\partial F_{i}(t)}{\partial I_{k}^{d}}=\left\{ \begin{array}{cc} (1-m)\beta_{i}(t)\frac{\theta_{i}(t)}{\mu_{B}H_{i}}\; & \text{if }k=i\;\\ mQ_{ik}\beta_{k}(t)\frac{\theta_{k}(t)}{\mu_{B}H_{k}}\; & \text{otherwise },\; \end{array}\right.$$

which can be rewritten in matrix form as

2.28 $$F^{\prime }=[(1-m)U_{n}+mQ]\beta (t)\theta (t)\frac{1}{\mu_{B}}N^{-1},$$

where **Q** is the matrix of the connection probabilities defined via the gravity model, ***β***(*t*) and ***θ***(*t*) are diagonal matrices whose non-zero terms are the exposure *β*_*i*_(*t*) and the shedding *θ*_*i*_(*t*) rates for each department *i*, and **N** is a diagonal matrix whose non-zero elements are the nodal populations *H*_*i*_. Let us now proceed with the computation of the basic reproduction number $R_{0}$ and the basic epidemicity index *e*_0_. In this case, the following properties hold: ***β***(*t*) = *β*_0_**U**_**n**_, ***θ***(*t*) = *θ*_0_**U**_**n**_, and, since at the beginning of the epidemic the infected compartment has an infinitesimal population and all other compartments are to be assumed as empty, **S** = **N**. The first two properties stem from equations (2.2) and (2.3), where the past disease prevalence and the number of WaSH interventions can be equalled to zero. As a consequence, matrix **F′** simplifies as

2.29 $$F^{\prime }=\frac{\beta_{0}\theta_{0}}{\mu_{B}}[(1-m)U_{n}+mQ]\;N^{-1}$$

and matrix **T**_**0**_ as

2.30 $$T_{0}=\omega [(1-m)U_{n}+mQ],$$

where *ω* = *σβ*_0_*θ*_0_/*μ*_*B*_. Letting

2.31 $$M=[(1-m)U_{n}+mQ],$$

we obtain $J_{0}^{⋆}=\omega M-\Phi U_{n}$ and **T**_**0**_ = *ω***M**. The basic reproduction number is thus given by

2.32 $$R_{0}=P(K_{0})=P(-T_{0}{\left(\Sigma_{0}\right)}^{-1})=\frac{1}{\Phi }P(T_{0})=\frac{\omega }{\Phi }P(M)=\frac{\omega }{\Phi }$$

because matrix **M** is row-stochastic, hence its spectral radius is equal to one. As for the basic epidemicity index,

2.33 $$e_{0}=P(H(\omega M-\Phi U_{n}))=\omega P\left(\frac{M+M^{T}}{2}\right)-\Phi ,$$

because the following property holds:

2.34 $$|H(J_{0}^{⋆}-\lambda U_{n})|=\left|\omega \frac{M+M^{T}}{2}-\Phi U_{n}-\lambda U_{n}\right|=\left|\omega \frac{M+M^{T}}{2}-\mu U_{n}\right|,$$

where μ=λ+Φ. By setting $\bar{\mu }=P((M+M^{T})/2)$, we obtain

2.35 $$e_{0}=\Phi \bar{\mu }R_{0}-\Phi .$$

The general case where we consider effective reproduction numbers and epidemicity indices cannot be solved analytically as matrix **T_t_** contains parameters ***β***, ***θ*** and the state variable **S** that are spatially and temporally heterogeneous. Let *ζ*_*t*_ = P(**T_t_**) = P(*σ***M*β***(*t*)***θ***(*t*)(1/*μ*_*B*_)**N**^−1^**S**) and *ε*_*t*_ = P(**H**(**T_t_**)). The following relation still holds:

2.36 $$e_{t}=\Phi \frac{\epsilon_{t}}{\zeta_{t}}R_{t}-\Phi .$$

This equation allows us to determine the threshold value that the reproduction number should take for the system to attain a stable, non-reactive disease-free equilibrium (*e*_*t*_ ≤ 0)

2.37 $$R_{t}\leq \frac{\zeta_{t}}{\epsilon_{t}}.$$

In the example of §4, the mean across all times of the ratio *ζ*_*t*_/*ε*_*t*_ is 0.729.

## 3. Results

**Figure 4.**
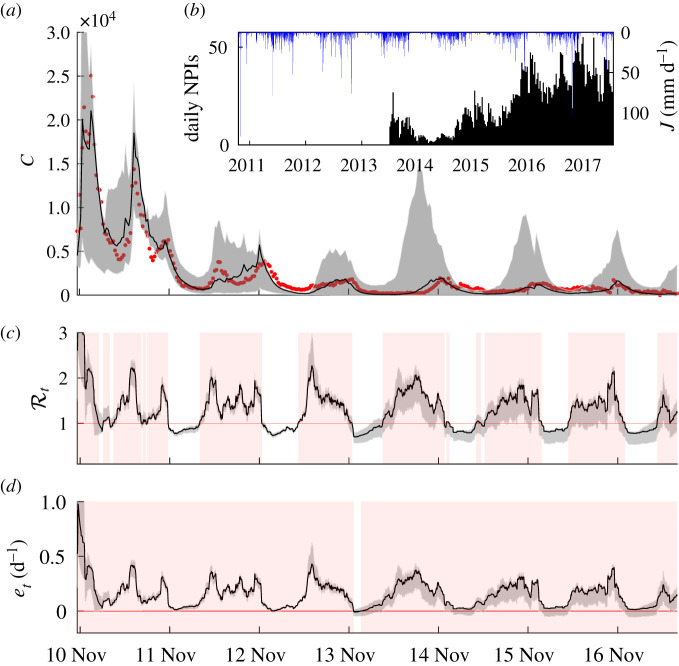
Temporal dynamics of disease incidence, effective reproduction number and epidemicity index. (*a*) Number of recorded (red dots) and simulated (black solid line) weekly infections in Haiti between October 2010 and June 2017. (*b*) Weekly NPIs deployed (grey bars) and incoming rainfall averaged over the whole Haitian territory (blue bars). (*c*) Effective reproduction number, smoothed over a four-week span. (*d*) Epidemicity index, also smoothed over a four-week span. Grey shading in (*a*), (*c*) and (*d*) denote the 50% confidence interval obtained by sampling several times the posterior distribution of the model parameters (electronic supplementary material). In (*c*,*d*), a red background indicates super-threshold values of either the effective reproduction number ($R_{t}>1$) or the epidemicity index (*e*_*t*_ > 0).

Figure 4 shows the computed time series of the two epidemiological indices introduced above, the effective reproduction number $R_{t}$ (§2.5) and the epidemicity index *e*_*t*_ (§2.6). The computed effective reproduction number exhibits frequent upcrossing of the unit threshold due to seasonal infection revamping via increased exposure to the disease brought in by washout of pathogens by tropical rainfall patterns. The epidemicity index *e*_*t*_ displays fluctuations synchronized to those exhibited by $R_{t}$, yet, critically, almost always inside a range of positive values. This indicates that dry seasons, albeit characterized by a sub-threshold $R_{t}$, still retain epidemic potential embedded in positive epidemicities. This, in turn, implies that a perturbation of the system by new infections may result in a possibly significant transient response by coalescence of sub-threshold local outbreaks. A linear regression between the epidemicity indices and the corresponding effective reproduction numbers reveals almost perfect correlation (*R*^2^ ≈ 1) and

**Figure 5.**
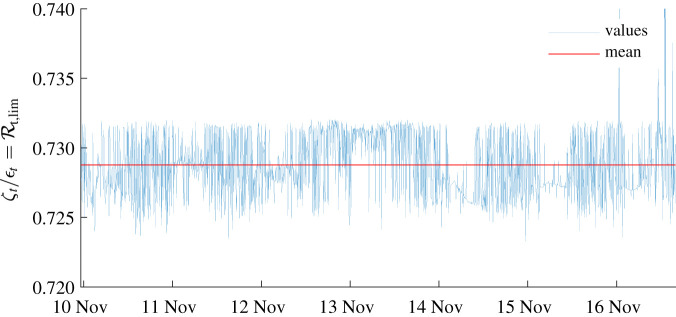
Time series of the temporal variation of the ratio between the spectral radius of the transmission matrix *ζ*_*t*_ and that of its Hermitian part *ε*_*t*_. The mean of the time series is shown in red. This ratio represents the upper bound of the effective reproduction number that would guarantee the effective epidemicity index to become negative, following the derivation shown in box 1.

3.1 $$e_{t}(t)=\beta_{0}+\beta_{1}R_{t}(t),$$

with coefficients *β*_0_ = −0.204 ± 0.01 [d^−1^] and *β*_1_ = 0.280 ± 0.01 [d^−1^]. It is interesting to note that, according to the attempted linear regression, $\beta_{0}=-\Phi =-(\mu +\alpha +\gamma )$ and $\beta_{1}=\Phi \bar{\epsilon_{t}/\zeta_{t}}$, the latter term corresponding to the average value of the ratio between the spectral radius of the Hermitian part of the transmission matrix and that of the transmission matrix itself (see box 1). Only small oscillations of the ratio around the mean can be observed, and this explains the very high correlation between $R_{t}$ and *e*_*t*_. The variation of the ratio is influenced by the evolution of the real-time epidemic and the changing values of parameters. Figure 5 displays the inverse of the ratio, which, as specified in box 1, represents the subthreshold value of the reproduction number that would guarantee a negative epidemicity index. This condition has been met only in a tiny temporal window (January 2014) as shown in figure 4*d*.

**Figure 6.**
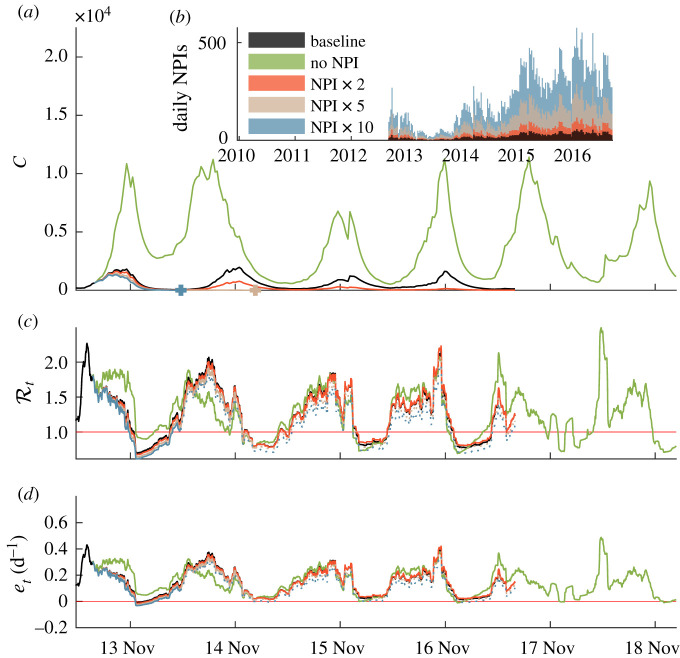
Unfolding of the epidemic and of the two key epidemiological indices for five different NPI deployment scenarios (baseline, no NPIs, two/five/10-fold NPI increase compared to baseline). (*a*) Simulated weekly infections (colour-coded as in (*b*)). Crosses represent the elimination of the corresponding epidemic scenario (*C*(*t*) < 1 onwards). (*b*) Daily NPIs being deployed Haiti-wide in the five considered scenarios. (*c*) Effective reproduction number. (*d*) Epidemicity index. Dotted lines in (*c*) and (*d*) represent the estimated indices after the elimination (see above) of the corresponding epidemiological trajectory. As data for NPIs are available up to July 2017, simulations generally end at the same time, except for the simulation related to the scenario with no NPIs, where we could simulate through January 2019. Data points before summer 2013 are hidden.

We tested alternative scenarios to assess the role of NPIs with regard to the time evolution of the number of infections and the two epidemiological indices $R_{t}$ and *e*_*t*_. As a benchmark, we considered a scenario where no pharmaceutical interventions were carried out in addition to the baseline scenario which implements the actual NPIs that have been carried out. Further additional scenarios were considered, corresponding to two-, five- and 10-fold increases in the number of NPIs with respect to the actual WaSH effort. Results are shown in figure 6. The scenario with no NPI led, as expected, to higher case incidence, effective reproduction numbers and epidemicity indices. This is a consequence of the higher exposure and shedding rates produced in the absence of NPIs. However, a higher disease incidence is, in turn, expected to induce stricter behavioural consequences limiting exposure for this scenario [3], as imposed by equation (2.2). This explains why the two considered epiemiological indices drop to values that are lower than those simulated in other scenarios after a high-incidence period, such as the one that might have been observed during summer 2014. Other scenarios, where the number of NPIs was increased with respect to the baseline, show a reduction in cholera incidence during the period of NPIs deployment, although only five- and 10-fold increases actually led to elimination (weekly detected cases *C* < 1) within the considered period (figure 6). However, even if the number of infected people has been brought to zero, the DFE might remain unstable and reactive, namely the increase in the number of NPI’s might be insufficient to avoid an epidemic caused by an injection of new cases, specially during periods with heavy rainfall. This can be ascertained by carrying on the calculation of $R_{t}$ and *e*_*t*_ even after the disease has been eliminated. As a matter of fact, figure 6 shows that this is indeed the case: $R_{t}$ and *e*_*t*_ are smaller than the values obtained in the other scenarios but can be above threshold under certain conditions.

**Figure 7.**
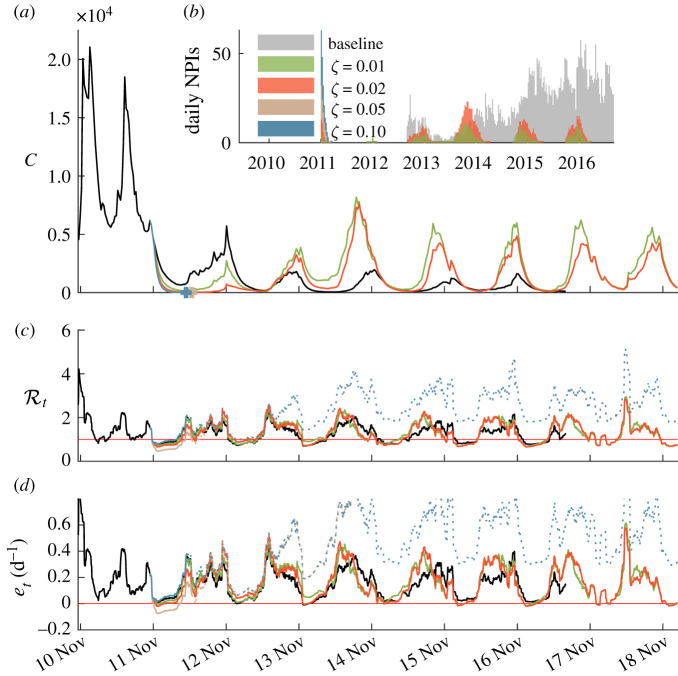
Unfolding of the epidemic and of the two key epidemiological indices for five different NPI deployment scenarios (baseline, number of NPIs proportional to weekly incidence with *ζ* ∈ {0.01, 0.02, 0.05, 0.1}). (*a*) Simulated weekly infections (colour coded as in (*b*)). Crosses represent the elimination of the corresponding epidemic scenario (*C*(*t*) < 1 onwards). (*b*) Daily NPIs being deployed Haiti-wide in the four considered scenarios. (*c*) Effective reproduction number. (*d*) Epidemicity index. Dotted lines in (*c*) and (*d*) represent the estimated indices after the elimination (see above) of the corresponding epidemiological trajectory. Once again, the baseline scenario stops in July 2017 due to data unavailability beyond that point.

An additional set of scenarios was created by linking the number of NPIs carried out with recent disease incidence. Specifically, we assumed that the number of NPIs deployed on a given day is proportional, via a factor *ζ*, to the total cholera incidence experienced during the previous week. Different effort levels have been foreseen, namely *ζ* ∈ {0.01, 0.02, 0.05, 0.1}, in addition to the baseline scenario reproducing what actually happened. We simulated these scenarios by assuming that the deployment of NPIs start on 1 November 2011, roughly 1 year into the 2010s Haitian cholera outbreak. The outcomes of these scenarios are detailed in figure 7. The two scenarios with *ζ* = 0.01 or *ζ* = 0.02 (one WaSH intervention every 100/50 weekly cases), after a first successful reduction in disease prevalence, led to additional infections with respect to the baseline scenario, as they are not vigorous enough to effectively eliminate the pathogen, and as the number of interventions dropped after a low incidence period, new epidemic waves take hold. The scenarios with *ζ* = 0.05 or *ζ* = 0.1 (one WaSH intervention every 20/10 weekly cases), instead, produced an effective response, and elimination would actually be achieved within the year in both cases. As progressively more and more interventions were deployed, depending on the recently recorded infections, cases would then be finally brought to near zero resulting in the extinction of the epidemic. However, since the number of NPIs is proportional to the number of cases, these scenarios imply a strong reduction of NPIs after the number of infected people has been brought near zero. This has the paradoxical effect of increasing the risk of a new epidemic, should an injection of pathogens occur, as shown by the above-threshold values of $R_{t}$ and *e*_*t*_ in figure 7 corresponding to the two scenarios. This result must be contrasted with the one of figure 6 where the number of implemented NPIs is maintained even after the disease elimination. In addition, when elimination is swiftly reached with a massive deployment of NPIs, a larger population of the population is left susceptible to infection, which contributes to the higher values of the two epidemiological indices.

**Figure 8.**
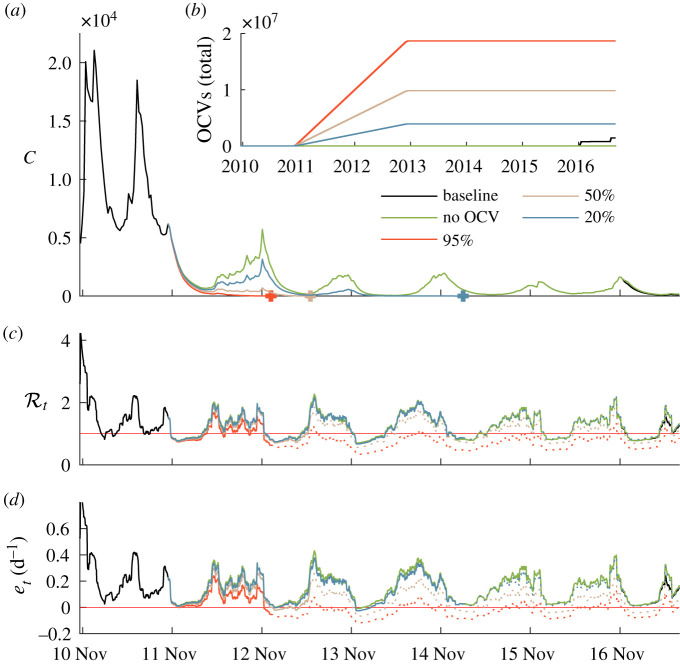
Unfolding of the epidemic and of the two key epidemiological indices for five different vaccine deployment scenarios (baseline, no vaccination, full vaccination for 95%, 50% and 20% of the population). (*a*) Simulated weekly infections (colour coded as in (*b*)). Crosses represent the elimination of the corresponding epidemic scenario (*C*(*t*) < 1 onwards). (*b*) Cumulative vaccine doses administered Haiti-wide in the five considered scenarios. (*c*) Effective reproduction number. (*d*) Epidemicity index. Dotted lines in (*c*) and (*d*) represent the estimated indices after the elimination (see above) of the corresponding epidemiological trajectory. We show our results through July 2017.

To disentangle the role of vaccination and NPIs towards cholera’s elimination in Haiti, we also developed additional scenarios to test a progressive vaccination of different quotas of the Haitian population, assuming a homogeneous vaccination across departments and compared these scenario with the actual vaccine rollout campaign. Specifically, we estimate that only 5.6% of the Haitian population has been vaccinated before July 2017 (some 878 173 first doses and 555 315 second doses given, see electronic supplementary material for additional information). Scenarios of vaccine deployment are shown in figure 8. According to the model, and to the temporal limitations thereof, the actual vaccination campaign in the considered period (November 2016 through June 2017, see electronic supplementary material) did not show any significant reduction in disease incidence or in the key epidemiological indices compared to a no-vaccination scenario. The effective reproduction number and the epidemicity index are slightly reduced by this vaccination effort. On the other hand, an earlier start of the campaign (say, 1 year after the outbreak) and a stronger effort (say, involving 20% of the total population) would have been much more effective in shortening the duration of the epidemic. More intense vaccination campaigns, potentially reaching 50% or 95% of the total population, would have obviously led to a faster elimination of cholera, as well as to lower values of the reproduction number and the epidemicity index.

## 4. Discussion

Epidemiological indices such as the effective reproduction number and the epidemicity index help guide the design of the deployment of control measures such as NPIs and vaccination campaigns. These indices can be computed through an array of different methods, typically stemming from the computation of the Jacobian matrix of the associated compartmental model [27,33,35,47]. In this respect, we dynamically computed the infectious subsystem and the related epidemiological indices. We found that, while the effective reproduction number fluctuates around its critical value ($R_{t}=1$), the epidemicity index does not, consistently remaining above its critical value (*e*_*t*_ = 0). Ascending or descending trends in the epidemicity index closely follow those of the effective reproduction number. Remarkably, we found (box 1) that these two indices are linearly related under the assumptions of this cholera model, owing to their common roots in the Jacobian matrix. This correlation thus sheds light on the value that the effective reproduction number should result in the sytem not only achieving a disease-free equilibrium condition asymptotically ($R_{t}<1$) but also avoiding transient amplifications of impulsive perturbations possibly leading to epidemic flare-ups (*e*_*t*_ < 0). According to this relationship, the epidemicity index becomes negative (i.e. the system ceases to be reactive) for values of the effective reproduction number that are on average

4.1 $$R_{t}\leq 0.729.$$

This represents a stricter bound for the reproduction number than the necessary crossing of the unit value. It could be used as a more ambitious goal for effective disease control. The epidemicity index therefore assumes a relevance beyond the assessment of control measures implemented to fight cholera in Haiti. Whether or not the extension to other infectious diseases is straightforward remains to be seen. Certainly, in the case of cholera and under the stipulations of the current model, this condition ultimately provides additional and safer guidance to policymakers engaged in the design of emergency interventions.

Although the 2010s Haiti cholera outbreak has now been declared extinct, different containment measures (such as NPIs and vaccination) have yet to be comparatively assessed. Since raw disease incidence is not a good short-term indicator as to whether a stable disease-free condition has been established yet, the computation of the effective reproduction number may provide a better understanding of the effectiveness of control measures. To that end, we evaluated the system’s response in terms of the disease incidence, effective reproduction number and epidemicity index under different scenarios of NPI and vaccine deployment. Our model also indicates that increasing the number of NPIs up to five/10-fold would have led to elimination of the disease from Haiti, whereas aggressively deploying NPIs from the onset of the epidemic (one intervention every 10 recorded infections) appears to be a more effective—in terms of the total number of interventions deployed—way of eliminating cholera. This result suggests that massively targeting new infections is indeed a good strategy, which would not only lead to elimination in a shorter time span, but also result in a lower number of total interventions deployed. This is somewhat similar to what has actually been done in Haiti with the colour-alert surveillance mechanism [9]. The importance of NPIs has also been suggested by [49]. They analysed the effect of WaSH interventions and vaccinations up to the actual elimination of the disease from Haiti. They argue that, since the vaccinations covered only a little part of the Haitian population and were rather concentrated in a few communities, WaSH interventions were indeed an efficient strategy to bring the outbreak to a halt. This argument is reinforced by the increasing value of the ratio of NPIs per detected case, in agreement with our findings. However, we remark that each deployed intervention directly acts on people’s exposure and on infectious shedding by reducing them for a given period. These reductions logically cause the number of new recorded infections to decrease, but also due to NPIs’ temporally limited effect, both the effective reproduction number and the epidemicity index end up increasing above the values they actually took in the baseline scenario, thus paving the way for a new outburst. WaSH interventions are therefore to be seen, from our scenarios, as temporary containment measures that efficiently reduce disease incidence in the short run. This is reinforced by the assumption that exposure depends on incidence-mediated awareness, and would therefore inevitably increase after a period characterized by a lower number of new recorded infections. However, since NPIs do not efficiently reduce the two considered epidemiological indices to sub-threshold values, without a comprehensive overhaul of the country’s water provisioning and sanitation infrastructure, they cannot guarantee possible revamping of the outbreak triggered by a reintroduction of the pathogen in Haitian waters. To conclude, WaSH interventions are useful actions that can powerfully drive the number of infections down if they start soon after the outbreak begins, are massively deployed across the population, and their distribution is maintained even beyond the peak of an epidemic wave, to properly ensure elimination. Ceasing the deployment of NPIs too early without having immunized a substantial portion of the Haitian population, and an early return to pre-epidemic conditions where no containment measures are taken by the policymakers nor the population could warrant a re-establishment if the pathogen is somehow reintroduced or has not been completely removed, which would lead to new infections, new death and new pressure on the healthcare and economic systems. However note that, in the proposed model, all NPIs have the same temporal duration, which might not be the case in reality. Some NPIs, especially those performed during periods of low incidence (such as construction toilets and water sanitation systems), might have a long-term impact and help lowering the successive transmission.

Vaccination may be a more effective tool to promptly eradicate a cholera epidemic. In this case, our model suggests that immunization of 20% of the Haitian population would have halted cholera transmission in approximately 2 years. The effective reproduction number and the epidemicity index would also have rapidly dropped to values that are lower compared to the baseline trajectory. In addition, as the vaccinated population would maintain a certain degree of acquired immunity for roughly 5 years after vaccine administration [37,50], a long-term reduction in the effective reproduction number and epidemicity index would have been guaranteed. NPIs, instead, are immediately effective but may only guarantee a temporary drop in these epidemiological indices. As a consequence, the country would be partially secured against any reintroduction of the pathogen only during this relatively brief interval. Our findings suggest that considering both the epidemicity index and the effective reproduction number may help rank (and target) containment interventions not only in the short- but also in the long term. Overall, we may conclude that only the enforcement of containment measures of epidemic cholera forcing negative values of the epidemicity index underpins a rapid elimination of an outbreak.

One additional key result of this study is the strong direct relationship between the reproduction number (either basic or effective) and the epidemicity index, which may hold true also for directly transmitted diseases. In fact, for the computations of the epidemiological indices, we introduced the assumption that the bacterial compartment is at equilibrium with (hence, proportional to) the infectious pool. Through this assumption, the mathematical description of cholera transmission dynamics becomes similar to that of directly transmitted diseases. Correlation analysis confirmed the theoretical predictions introduced in box 1. The implications of this result are noteworthy. While the necessary condition of attaining an effective reproduction number less than unity sheds light on the feasibility, in the long run, of reaching an asymptotically stable disease-free equilibrium, it does not provide any sufficient information to prevent transient blooming responses and the coalescence of sub-threshold outbreaks. This is a direct consequence of the analysis of whether the infectious subsystem retains its reactivity. The definition of a linkage between the two indices shows that the disease reproduction number must necessarily drop to a well-defined value lower than unity to prevent the system from being reactive and possibly temporarily amplify transient perturbations. As a consequence, policymakers should consider additional, stricter bounds for $R_{t}$ when designing their interventions. This may lead to a better and more informed deployment of containment measures, such as vaccinations or NPIs. In addition, a linkage between the disease reproduction number and the epidemicity index would allow one to compute the latter as a function of the former. While methods to compute the disease reproduction number without calibrating a compartmental model, and directly from incidence data exist e.g. [20,25], the epidemicity index does not have a direct computation available to date.

A limitation of this study is certainly represented by the incomplete availability of epidemiological data regarding NPIs, which prevented us from carrying out our analyses across the entire Haitian cholera outbreak. Therefore, it is not possible for us to fully disentangle the mutual effectiveness of vaccinations and NPIs towards the elimination of the Haitian cholera outbreak. This study could be further improved by considering a variety of models for NPIs and formally comparing them through statistical indicators such as the Akaike information criterion [51].

## 5. Conclusion

The following conclusions are worth mentioning:

— a deterministic, spatially explicit mathematical model of epidemic cholera has been fitted with all the tools needed to account for an array of containment measures. We have revisited the stability of the disease-free equilibrium, identifying indices that characterize either long- or short-term epidemic behaviour (basic and effective reproduction numbers, $R_{0}$ and $R_{t}$, versus epidemicity indices, *e*_0_ and *e*_*t*_). Properly calibrated, the model and its related indices show a keen ability to reproduce observed epidemiological space–time patterns in the notable case of the Haiti 2010–2019 epidemic;
— the evaluation of alternative intervention scenarios allowed us to rank NPIs and vaccine deployment strategies in terms of their effectiveness towards elimination of the disease;
— the enforcement of containment measures of epidemic cholera is effective in prompting disease elimination only if forcing negative values of the epidemicity index. Negative epidemicity in fact warrants a rapid elimination of an outbreak, differently from effective reproduction numbers below the unit threshold when epidemicity values remain positive;
— we found that in cholera compartmental models, under a mild set of assumptions, the effective reproduction number and the epidemicity index are related through a linear relationship. Thus we may identify the upper bound (significantly lower than 1) for the former to warrant a negative value of the latter. This result suggests new avenues for the design of emergency containment measures.

## Data Availability

All codes implemented to develop the computations of this study and the figures thereof are provided in the GitHub repository available at https://doi.org/10.5281/zenodo.5961699 [52].
